# Evaluation of Selected Fire Properties of Recycled Particleboards

**DOI:** 10.3390/polym17060805

**Published:** 2025-03-18

**Authors:** Zuzana Vidholdová, Martin Zachar, Ján Iždinský, Viktória Satinová

**Affiliations:** 1 Department of Wood Technology, Faculty of Wood Sciences and Technology, Technical University in Zvolen, Masaryka 24, 96001 Zvolen, Slovakia; jan.izdinsky@tuzvo.sk (J.I.); vsatinova@gmail.com (V.S.); 2 Department of Fire Protection, Faculty of Wood Sciences and Technology, Technical University in Zvolen, 96001 Zvolen, Slovakia; zachar@tuzvo.sk

**Keywords:** burning rate, fire protection, ignition time, mass loss, particleboard

## Abstract

This study evaluated the fire properties of various particleboard (PB) types, including those made from sound spruce particles, degraded wood (brown and white rot), and recycled materials (blockboard, pallets, thermally modified wood, raw and laminated PBs, or mixtures). Laboratory-manufactured PBs showed densities ranging from 587 to 654 kg·m^−3^, with higher densities generally correlating with longer ignition times, although no statistically significant relationship was observed. Ignition times varied depending on material composition, with PBs made from sound spruce particles exhibiting the longest ignition times (103 ± 4.89 s). In contrast, PBs containing recycled or degraded particles ignited faster, influenced by additives such as adhesives and laminates. The burning rate peaked between 90 and 180 s, with PBs containing recycled raw PB particles and those degraded by brown rot showing the highest maximum burning rates (0.214 and 0.213 %·s^−1^, respectively). Recycled laminated PBs reached peak burning rates in the shortest time, while control PBs required the longest time. Mass loss was higher in PBs with recycled or degraded particles, ranging from 47.52% to 51.71%, compared to 44.89% for control PBs. These findings highlight the trade-offs between fire resistance and the use of recycled materials, emphasizing the impact of additives on combustion behavior.

## 1. Introduction

Particleboards (PBs) are commonly used in furniture manufacturing, construction, and interior design due to their cost effectiveness and efficient use of wood resources [1,2,3]. We can observe a more or less steady increase in their production over the last ten years, with their world production exceeding 116 million cubic meters in 2023 [4].

The rising production of PBs related to the growing demand also requires an ever-increasing amount of input raw materials for the production of PBs, and more attention is being focused on developing innovative materials based on natural resources [5]. As input raw materials from which wood particles (particle) are produced for the production of PBs, it is possible to use natural wood, or degraded damaged wood of all wood species occurring in Central Europe, secondary wood raw materials from forestry, post-industrial wood residues (e.g., forest residues, chips, off-cuts, shavings, and sawdust), lignocellulosic raw materials (e.g., bagasse, flax and hemp shives, etc.), or recycled wood (post-consumer sources of recovered wood—e.g., old furniture, demolition wood, wood-based panels, pallets, boxes, solid wood packaging) as a suitable raw material for the industrial production of wood composites [6,7]. Low-quality wood and especially recycled wood are currently most widely used when sufficient quantities are available for the production of PB, which supports the principle of a circular economy. The recycled wood proportions in particleboard vary by country: ≈100% in Italy; ≈50% in Belgium, the United Kingdom, and Denmark; and 15–30% in Germany, France, and Spain [8,9,10]. The presence of diverse input raw materials affects the properties of PB, including mechanical, physical, biological, technological, and fire-resistance properties, which determine its suitability for certain applications.

Fire resistance is a crucial property when evaluating PBs for applications where safety under high temperatures is a concern. The flammability of PBs depends on multiple factors, including material composition, density, and the presence of additives [11,12,13,14,15,16]. Parameters such as ignition time, burning rate, and final mass loss provide valuable insights into the combustion behavior of PBs. The use of recycled materials or degraded wood may affect these parameters due to differences in density, particle composition, and chemical additives introduced during manufacturing. Despite its significance, the reaction-to-fire performance of recycled PBs remains an underexplored area in the literature.

Popescu and Pfriem [17] provide a comprehensive overview of existing fire-retardant and intumescent coating materials, modifications, and treatments that can be applied to wood and wood-based products to improve their fire performance, where fire-retardant solutions are used to improve their fire-resistance properties. These are based on compounds that interfere with a certain stage of the combustion process and contain halogens (chlorine or bromine), phosphorus, nitrogen, boric acid, silicon, hydroxides, phosphates, carbonates, sulphates, or inorganic metal compounds [18]. Traditionally, three vital perspectives were taken into consideration for a chemical to be used as a fire retardant: the kind and amount of toxic gases that are produced, the extent to which the mechanical properties are reduced, and its effects on the hygroscopicity [19].

Several studies have explored the fire resistance of PBs composed of or incorporating agricultural residues such as Canary Island palm trunks [18], oil palm trunks from Malaysia [20], kenaf [21], flax [22], hemp shives [23], rice straw [24], and rice husk [25]. Taghiyari et al. [19] investigated the fire properties of PB without and with 5 and 10% feather content, to which 10% wollastonite was added to reduce the flammability of the chicken feathers. They found that the chicken feather content of 5% seemed to be a good option to partially satisfy the ever-growing need for natural materials, and to keep the fire properties of panels at an acceptable level as well. Suh et al. [26] investigated the fire-resistance characteristics of recycled particle boards, the particles of which were treated with fire-retardant chemicals (dibasic ammonium phosphate and boric acid). Fire-resistance tests and cone calorimeter tests were conducted for thick plywood, particleboard, and medium-density fiberboard with sample thicknesses of about 28–30 mm, and their suitability for quasi-fireproof or fire-preventive structures was evaluated [27].

Despite these advances, further research is needed to understand the influence of different raw materials on the fire performance of PBs, particularly those incorporating degraded or recycled wood. Addressing this gap is crucial for improving fire safety and optimizing the use of alternative materials in PB production.

This study investigates the fire properties of PBs produced from sound spruce wood, degraded wood (brown and white rot), and various recycled materials. By examining ignition time, burning rates, and mass loss, this research provides valuable understandings into how material composition influences the flammability of PBs, contributing to safer and more sustainable PB production practices.

## 2. Materials and Methods

### 2.1. Particleboard Variants

The type P2 three-layer particleboards (PBs) for interior use were manufactured from the following wood particles:

(I) Spruce-derived particles, obtained from spruce particles in different conditions: (a) sound wood, (b) wood degraded by brown rot, and (c) wood degraded by white rot. The quantity of brown and white rot in timber is analyzed in [28].

(II) Recycled wood materials, including (a) blockboard, (b) aged spruce pallets, (c) off-cuts of thermally modified wood, (d) unlaminated PBs removed from production, (e) laminated PBs, and (f) a mixture of recycled wood materials.

The PBs with the dimension of 400 × 300 × 16 mm were manufactured under laboratory conditions, as was mentioned in previous studies [29,30,31,32]. A total of 6 pieces of board were manufactured for each type. Each type of PB was manufactured with 100% amounts of sound, rotten, or recycled particles. The particles were bonded with UF glue containing additives “hardener and paraffin” in the recipe, used at KRONOSPAN s. r. o, Zvolen, Slovakia. The glue mixture was applied to the particles in a rotary mixing machine (VDL, TU, Zvolen, Slovakia). The particles layered with glue were then cold-pressed in a low-temperature machine at a pressure of 1 MPa in a CBJ 100-11 press (TOS, Rakovník, Czech Republic) according to a three-stage pressing diagram at a maximum pressing plate temperature of 240 °C, a maximum specific pressing pressure of 5.23 MPa, and with a pressing factor of 8 s·mm^−1^. The experiment also utilized commercial three-layer PBs.

### 2.2. Method of Determination of Density of Particleboards

Test specimens with the dimension of 50 × 50 × 16 mm of all variants of PBs were conditioned in a climate chamber (type Memmert, model 410, Schwabach, Germany) for two months. The chamber maintained environmental conditions of 22 ± 2 °C and a relative humidity of 65 ± 5%. The density of the PBs was measured according to EN 323 [33].

### 2.3. Methods of Determination of Fire Properties

**Figure 1:**
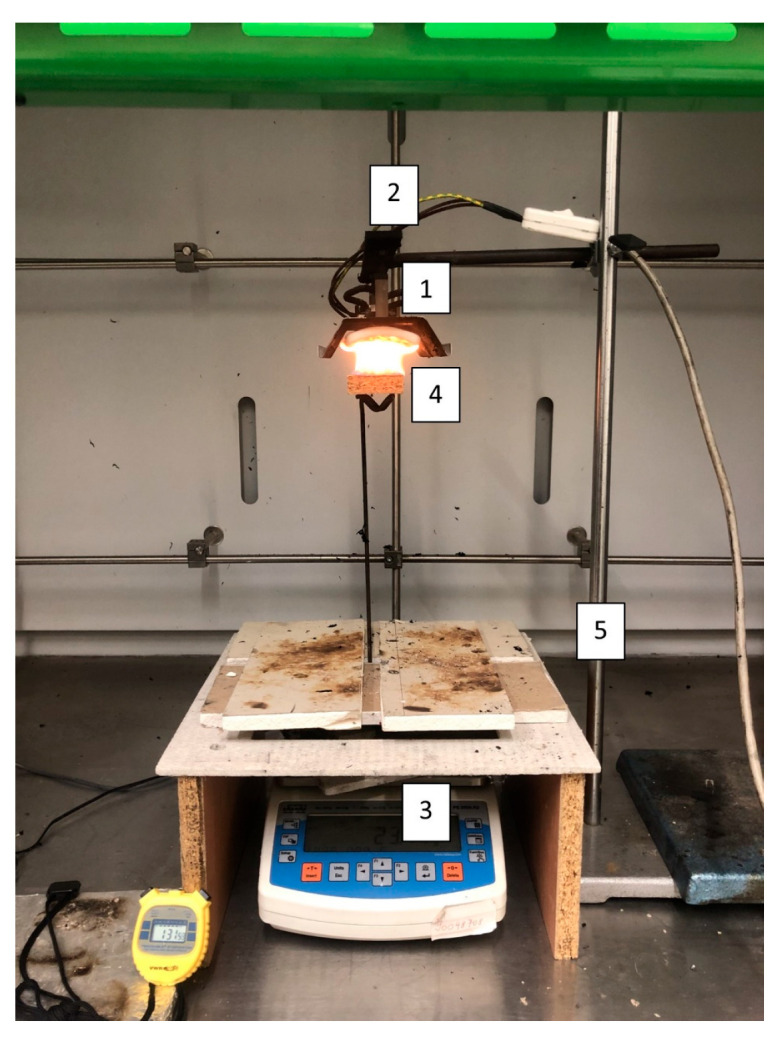
The experimental setup for testing with a radiant heat source consisting of the following components: (1) ceramic infrared heater, (2) supporting metal frame for positioning the radiant heat source, (3) accurate digital scales, (4) designated placement area for test specimens, and (5) metal stand for securing and stabilizing the setup.

As a non-standard test method, a radiant heat source test was chosen to evaluate the fire properties of the examined recycled PB samples. Test specimens with the dimension of 50 × 50 × 16 mm were thermally loaded by a heat flux of 30 kW·m^−2^ using a ceramic infrared heater with a power of 1000 W. The apparatus is shown in Figure 1. The sample was positioned in the holder at a distance of 30 mm from the ceramic infrared heater for a specific duration of 600 s. The weight change was recorded every second.

The evaluation of the fire properties was based on the following criteria:

- Ignition time (the time required for the test specimen to ignite);
- Burning rate—over time and maximum value (the rate at which the material combusts under thermal load);
- Mass loss—over time and at the end of test (the reduction in the sample’s mass during thermal exposure).

The burning rate was determined using the following equation:

(1) $$\vartheta =\frac{\delta \left(\tau \right)-\delta \left(\tau +\Delta \tau \right)}{\Delta \tau },$$

where ϑ—burning rate (%·s^−1^); δ(τ)—mass loss at time τ (%); δ(τ + Δτ)—mass loss at time τ + Δτ (%); Δτ—time interval for mass measurement(s).

The relative mass loss over time was calculated using the following equation:

(2) $$\delta_{m}\left(\tau \right)=\frac{m\left(\tau_{0}\right)-m\left(\tau \right)}{m\left(\tau_{0}\right)}\cdot 100,$$

where δ_m_—relative mass loss over time [τ = 0–600 s] (%); m(τ_0_)—mass of the sample at time τ = 0 (initial time, 0 s) (g); m(τ)—mass of the sample at time τ (g).

### 2.4. Statistical Evaluation

Descriptive statistics, including the median, mean, and standard deviation, were computed for the measured properties. The experimental data were analyzed using STATISTICA 12 software (StatSoft, Inc., Tulsa, OK, USA).

## 3. Results and Discussion

The results of the experiments evaluating the response of all types of PB samples (commercial, spruce-derived particles (sound wood, wood degraded by brown rot or white rot) and recycled wood materials (blockboard, pallets, thermally modified wood, raw or laminated PBs, or a mixture of recycled materials)) to the exposure to a certain degree of heat flux of the wood are summarized in Table 1.

### 3.1. Density of Particleboards

The density of laboratory-manufactured PBs in a conditioned state, manufactured from either sound spruce particles, low-quality wood particles degraded by fungal decay, or various recycled material variants (blockboard, pallets, thermally modified wood, raw or laminated PBs, or a mixture of recycled materials), ranged from 587 to 654 kg·m^−3^. The density of commercial PBs and laboratory control PBs manufactured from sound spruce wood was identical, at 654 kg·m^−3^. However, reduced densities were observed in PB variants containing particles derived from pallets, thermally modified wood, unlaminated PBs, and recycled material mixtures. These differences likely result from the manual layering of particles during PB manufacturing.

The density of PBs significantly influences their fire-technical properties. In study [34], it was reported that medium-density fiberboard (MDF) and PB, which have relatively higher densities, exhibited longer ignition times compared to oriented strand board (OSB) and plywood. This effect was attributed to the higher surface density of the material, which delays the attainment of ignition temperature at the surface and reduces the release of volatile gases necessary for ignition. Similarly, study [35] demonstrated a positive correlation between increased density and improved fire resistance. Therefore, optimizing the density of PBs is an important factor in enhancing their fire performance.

### 3.2. Evaluation of Fire Properties of Particleboards Under Radiant Heat

**Figure 2:**
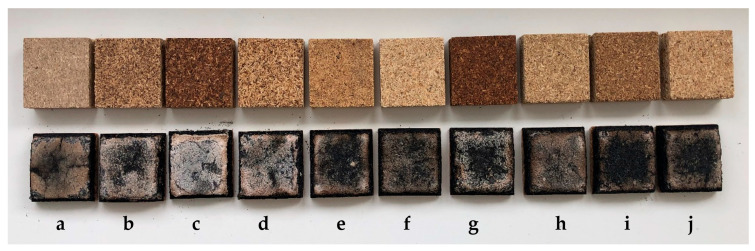
Samples of PBs before (**upper row**) and after (**lower row**) heat testing (50 × 50 × 16 mm, length × width × thickness): (**a**) PB—commercial, (**b**) PB—sound spruce wood, (**c**) PB—wood degraded by brown rot, (**d**) PB—wood degraded by white rot, (**e**) PB—blockboard, (**f**) PB—aged spruce pallets, (**g**) PB—thermally modified wood, (**h**) PB—unlaminated PBs, (**i**) PB—laminated PBs, and (**j**) PB—a mixture of recycled wood materials.

The radiant heat test, a non-standard method for evaluating material behavior under high temperatures, was applied to PB samples. Figure 2 illustrates the appearance of PB samples before and after testing.

#### 3.2.1. Influence of Density on the Ignition Time

**Figure 3:**
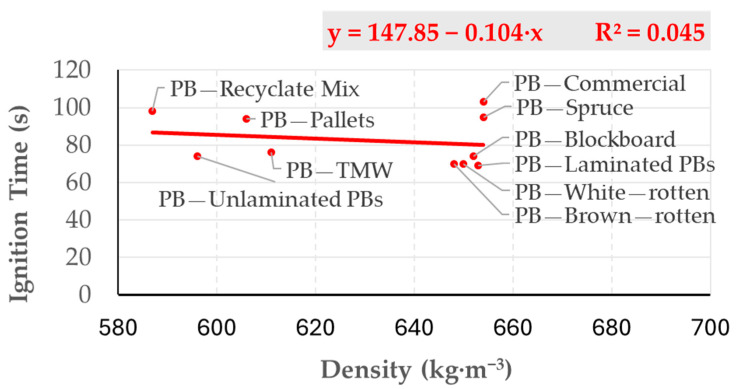
Relationship between the particleboards’ density and ignition time.

Under a thermal load generated by a radiant heater (1000 W, approximately 30 kW·m^−2^, positioned 30 mm from the sample surface), the ignition time for laboratory-produced control PBs (654 kg·m^−3^, sound spruce wood) was 103 ± 4.89 s. For other PB variants, the ignition times ranged from 69 to 98 s (Table 1). No statistically significant correlation between PB density and ignition time was observed (Figure 3).

Achieving longer ignition times is critical for enhancing fire resistance. PBs made from less processed solid wood (e.g., sound spruce logs, pallet boards, or recycled material mixtures) demonstrated the longest ignition times, highlighting the importance of material composition and processing methods.

Comparable results have been reported in prior studies. Study [36] investigated the effect of heat flux intensity (ranging from 43 to 50 kW·m^−2^) and ignition temperature on the combustion behavior of PBs and OSBs. Their study reported an ignition time between 60 and 117 s for PBs, with PBs exhibiting a shorter ignition time compared to OSBs. This difference is attributable to the structural composition of the boards, as OSBs are manufactured from larger wood strands than PBs. Study [35] observed even shorter ignition times of 34 s for 15 mm thick PBs. The observed variations in ignition times for solid materials are influenced by factors such as wood species, the performance characteristics of the radiant panel, heat flux intensity, and the distance from the heat source [37]. The specific chemical compositions of adhesives and laminates could influence their fire performance. UF adhesive exhibits lower thermal stability compared to PF (phenol–formaldehyde) and MDI (methylene diphenyl diisocyanate) adhesives, which show better thermal stability and higher char yield at 850°C. UF adhesive begins to decompose at a lower temperature (127 °C) compared to PF and MDI adhesives, which decompose at higher temperatures. The concentration of evolved gases (e.g., CO_2_, NH_3_) is higher for UF adhesive, indicating more significant emissions during thermal degradation. PF adhesives are considered more environmentally friendly due to lower emissions of toxic gases during combustion. These factors contribute to differences in ignition time and burning behavior among the adhesives and laminates [38].

#### 3.2.2. Influence of the Density and Material Type on the Burning Rate

The absolute burning rate, expressed in percent per second (%·s^−1^), measures how rapidly a material loses mass under thermal load. Materials with higher burning rates pose greater safety risks due to their faster combustion, while lower rates indicate safer, slower-burning materials. However, as noted [13,34,39], the ignition and combustion behavior of particleboard are challenging to analyze due to its inhomogeneous structure, void spaces, density variations, and the continuous charring process.

**Figure 4:**
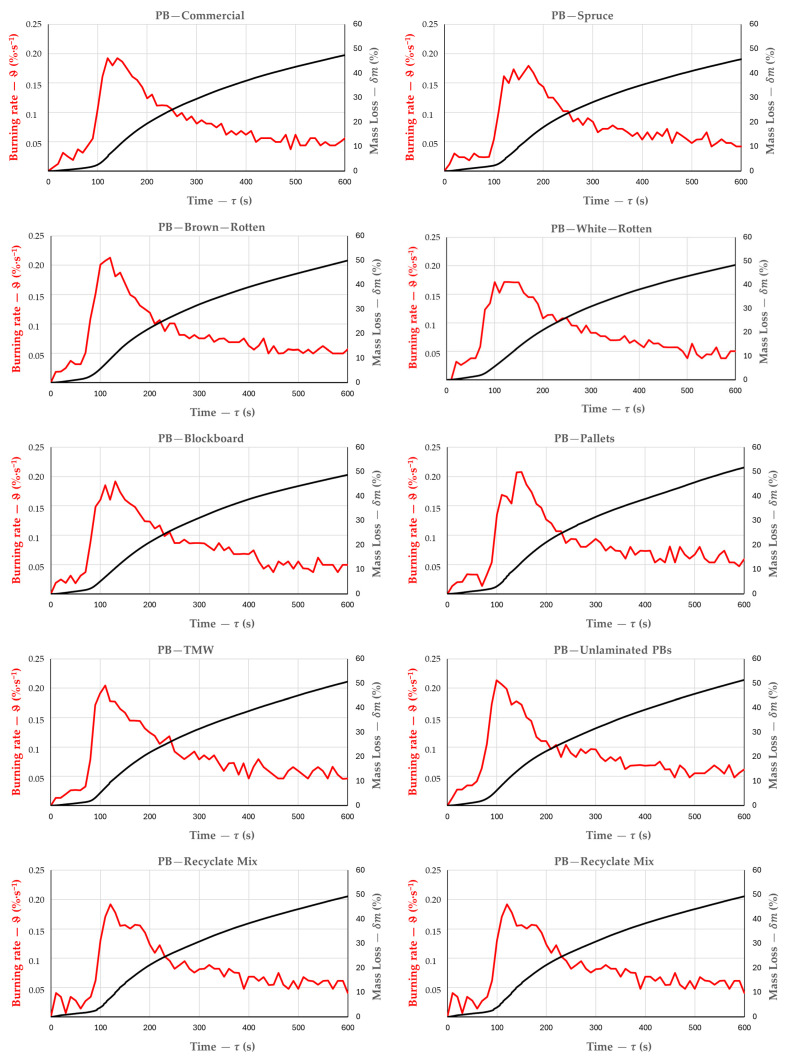
Average burning rate and mass loss over time for particleboards.

The progression of the burning rate over time was similar across PB types (Figure 4). The burning rate increased rapidly during the initial phase of the test, peaking between 0.17 and 0.22%·s^−1^ at approximately 100 s. The most rapid burning occurred between 90 and 180 s, corresponding to the first quarter of the test. After peaking, the rate declined gradually and stabilized, with smaller fluctuations around 200–300 s.

The time to reach the maximum burning rate is a key parameter for fire resistance. PBs made from recycled raw PB particles reached the highest burning rate (see Table 1; 0.214%·s^−1^ at 100 s), followed by PBs containing brown-rot-degraded particles (0.213%·s^−1^ at 120 s). Conversely, PBs made from white-rot-degraded particles exhibited the lowest maximum burning rate (0.172%·s^−1^ at 130 s). PBs made from recycled laminated particles reached their peak rate the fastest (90 s), while laboratory-produced reference PBs from sound spruce wood took the longest (180 s).

Additives from recycled materials (e.g., adhesives, laminates) reduced both the time to reach the maximum burning rate and ignition time, underscoring their influence on combustion behavior. Using minimally processed solid wood particles extended the time to peak burning rate, emphasizing the impact of material selection on fire-technical properties.

Further, the pattern of peak burning rates observed between 90 and 180 s across the different PB types was influenced by a combination of material composition, density, moisture content, adhesive formulation, surface oxidation, and charring behavior. Variations in these factors affect the combustion dynamics and thermal degradation pathways of PBs, leading to differences in burning rate progression. The composition of PB significantly impacts its combustion behavior. PBs manufactured from degraded wood, particularly those affected by brown rot, exhibit increased combustibility due to cellulose degradation, which enhances volatile release and accelerates burning. In contrast, PBs composed of white-rot-degraded wood exhibit a lower peak burning rate, as lignin decomposition leads to a more porous char structure that moderates oxidation. Additionally, PBs incorporating recycled wood materials demonstrate heterogeneous combustion behavior due to variations in feedstock properties and adhesive residues. Density and porosity further influence burning dynamics. Lower-density PBs, such as aged spruce pallet PB, facilitate greater oxygen penetration, leading to a rapid increase in the burning rate. Conversely, higher-density PBs, such as laminated PBs, delay peak burning due to restricted oxygen diffusion and reduced heat transfer. Moisture content also plays a crucial role, as PBs with lower moisture levels, such as thermally modified and aged PBs, reach peak burning more rapidly due to reduced heat absorption and faster volatile release [37]. Surface oxidation significantly contributes to combustion intensity by governing the rate of oxygen uptake. PBs with high oxidation potential, such as unlaminated and brown rot PBs, exhibit faster combustion due to increased surface reactivity and enhanced oxygen diffusion. In contrast, laminated PBs and those with protective coatings exhibit reduced oxidation rates, delaying peak combustion. Additionally, charring behavior affects burning progression, with PBs forming stable, insulating char layers, such as in sound spruce PB, exhibiting slower burning rates due to limited heat penetration. In contrast, PBs with weak or fragmented char layers, such as thermally modified and unlaminated PBs, sustain higher burning rates due to reduced thermal resistance [13,37].

#### 3.2.3. Influence of Particleboard Type on the Final Mass Loss

**Figure 5:**
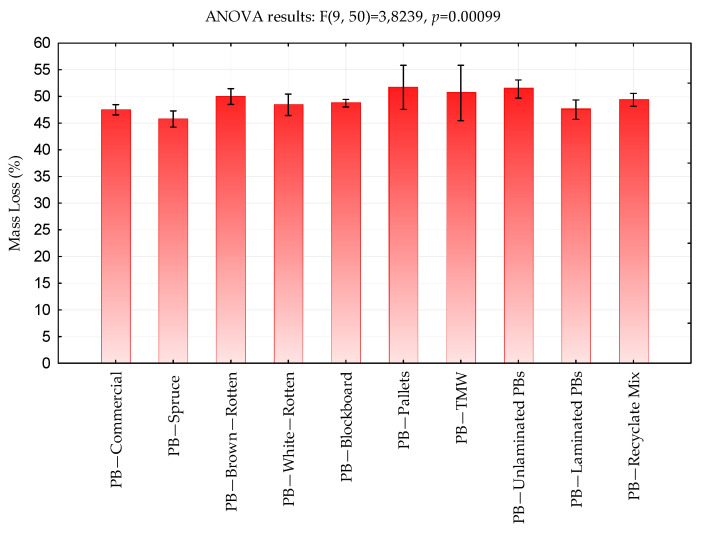
Average final mass loss of particleboards.

The mass loss of the PBs increased steadily over time, with no significant decline (Figure 4). PB variants containing degraded particles or recycled materials exhibited final mass losses ranging from 47.52% to 51.71% after 600 s—up to 7% higher than laboratory control PBs made from sound spruce wood (Table 1, Figure 5). Particleboards made from untreated spruce and other deciduous wood particles generally exhibit slightly poorer fire resistance, with mass loss exceeding 50%, depending on board thickness, material density, particle composition, adhesive type, moisture content, presence of fire retardants, surface treatment, particle geometry, and exposure conditions [3,5,35,36,40].

**Figure 6:**
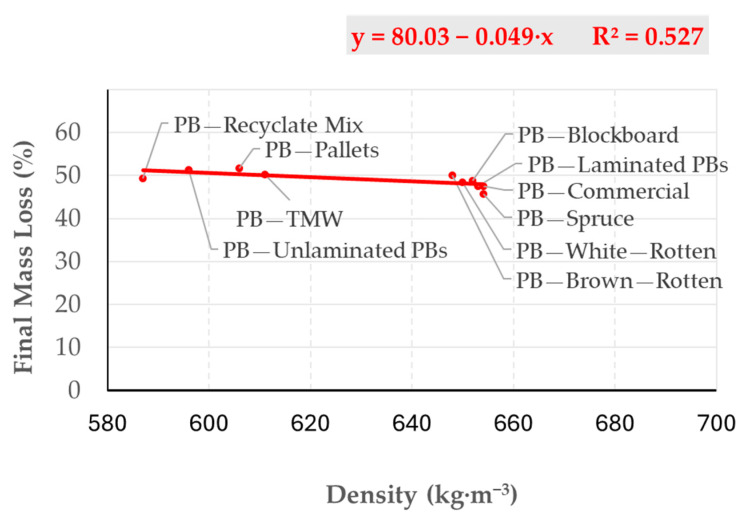
The dependence of mass loss on density of particleboards.

Particleboards (PBs) with reduced density, particularly those incorporating particles from pallets, thermally modified wood, or recycled material mixtures, exhibited increased mass loss when exposed to radiant heat (Figure 6). Pre-treatment processes applied to raw materials during the preparation of particles from recycled, thermally modified, or decayed wood—such as trimming or grinding—reduce particle length to the desired size, as demonstrated by [28,31] through sieve analysis. Variations in particle geometry significantly influence the overall mass loss of PBs [41].

The mass loss analysis of PBs made from degraded materials being higher than that of sound wood might be linked to the complex interactions between the degraded particles and the adhesives or other additives used in manufacturing. Since fungal degradation does not necessarily produce a uniform or deep breakdown [42], the exposed surface of these boards may contribute more readily to combustion in the early stages of burning, leading to an initial increase in mass loss. However, as the surface chars and becomes less reactive, the rate of mass loss stabilizes. This could be also an explanation for the steady increase in mass loss over time without significant decline.

## 4. Conclusions

This study evaluated the fire properties of various particleboard (PB) types, including those produced in the laboratory from sound spruce particles, wood degraded by fungal decay (brown rot and white rot), or recycled wood materials (blockboard, pallets, thermally modified wood, raw or laminated PBs, and material mixtures), and commercially produced PBs.

Laboratory-produced PBs with the highest density (654 kg·m^−3^) exhibited the longest ignition times, emphasizing the role of density in enhancing fire resistance. However, no statistically significant correlation between density and ignition time was observed, suggesting that other factors, such as material composition and processing methods, play a critical role.

The burning rate progression showed a rapid increase during the initial phase, peaking between 90 and 180 s, followed by a gradual decline. PBs containing recycled materials and degraded particles (e.g., brown rot) reached their maximum burning rate faster than PBs made from sound spruce wood. Additives from recycled materials, such as adhesives and laminates, significantly influenced the combustion behavior by reducing both the time-to-peak burning rate and the ignition time.

Final mass loss was highest in PBs containing recycled and degraded particles, ranging from 47.52% to 51.71%, compared to 44.89% in control PBs made from sound spruce wood. PBs with lower densities exhibited increased mass loss, further highlighting the impact of recycled content on fire performance.

PBs incorporating minimally processed solid wood particles, such as sound spruce logs and pallet boards, demonstrated enhanced fire resistance, with longer ignition times and delayed maximum burning rates. This underscores the importance of material selection in optimizing PB fire-technical properties.

Overall, the findings suggest that incorporating recycled materials into PBs introduces chemical additives that can reduce fire resistance, while using less processed solid wood particles improves fire performance. These results highlight the importance of material selection and processing in optimizing the fire-technical properties of PBs. In particular, PBs made from sound wood or minimally processed materials, such as spruce logs and pallet boards, demonstrated superior fire resistance with longer ignition times and delayed peak burning rates. Future research should focus on optimizing PB composition to achieve an ideal balance between fire safety, sustainability, and cost effectiveness for various applications.

## Figures and Tables

**Table 1:** Summary of particleboards’ fire properties.

Type of PBs	Density		Ignition Time		Mass Loss		Maximum Burning Rate	
	$\bar{x}$	SD	$\bar{x}$	SD	$\bar{x}$	SD	Time	Burning Rate
PB—Commercial	654	9	95	7.17	47.48	0.91	140	0.192
PB—Sound Spruce Wood	654	22	103	4.89	45.76	1.63	180	0.180
PB—Wood Degraded by Brown Rot	648	29	70	6.05	50.00	1.41	120	0.213
PB—Wood Degraded by White Rot	650	35	70	7.17	48.44	1.91	130	0.172
PB—Blockboard	652	25	74	5.85	48.74	0.67	130	0.192
PB—Aged Spruce Pallets	606	17	94	6.11	51.71	3.95	150	0.208
PB—Thermally Modified Wood	611	13	76	4.68	50.26	5.24	110	0.205
PB—Unlaminated PBs	596	30	74	9.32	51.39	1.62	100	0.214
PB—Laminated PBs	653	29	69	4.55	47.52	1.74	90	0.182
PB—Mixture of Recycled Wood Materials	587	22	98	3.14	49.38	1.15	120	0.192

Note: $\bar{x}$ —average, SD—standard deviation.

## Data Availability

The raw data supporting the conclusions of this article will be made available by the authors on request.
